# Return to Sport, Reinjury Rate, and Tissue Changes after Muscle Strain Injury: A Narrative Review

**DOI:** 10.1155/2024/2336376

**Published:** 2024-09-04

**Authors:** Mette W. Wulff, Abigail L. Mackey, Michael Kjær, Monika L. Bayer

**Affiliations:** ^1^ Institute of Sports Medicine Copenhagen Department of Orthopedic Surgery M Copenhagen University Hospital-Bispebjerg and Frederiksberg, Copenhagen, Denmark; ^2^ Center for Healthy Aging Department of Clinical Medicine Faculty of Health and Medical Sciences University of Copenhagen, Copenhagen, Denmark

## Abstract

A major challenge in sports medicine is to facilitate the fastest possible recovery from injury without increasing the risk of subsequent reruptures, and thus effective rehabilitation programs should balance between these two factors. The present review focuses on examining the role of different resistance training interventions in rehabilitation of acute muscle strain in the time frame from injury until return to sport (RTS), the rate of reinjuries, and tissue changes after injury. Randomized, controlled trials dealing with a component of resistance training in their rehabilitation protocols, as well as observational studies on tissue morphology and tissue changes as a result to muscle strain injuries, were included. The mean time for RTS varied from 15 to 86 days between studies (*n* = 8), and the mean rate of reinjury spanned from 0 to 70%. Eccentric resistance training at long muscle length and rapid introduction to rehabilitation postinjury led to significant improvement regarding RTS, and core-stabilizing exercises as well as implementing an individualized algorithm for rehabilitation seem to reduce the risk of reinjury in studies with a high rerupture rate. Independent of the rehabilitation program, structural changes appear to persist for a long time, if not permanently, after a strain injury.

## 1. Introduction

Acute muscle overload injuries, in terms of strains or ruptures, are some of the most prevalent sport injuries among both professional and amateur athletes [1, 2]. These injuries are typically a consequence of explosive movements or from stretching the muscle to extreme lengths. They are collectively referred to as “indirect” muscle injuries, as opposed to “direct” muscle injuries which comprise contusions and lacerations [3, 4]. In sports that entail running and other extensive use of the lower extremity, indirect muscle strains are primarily observed in the hamstrings but are also frequent in the calf or quadriceps [1, 4]. These injuries can be classified based on their severity, ranging from grades 1–3 or grades 1–4, depending on the classification system applied. The range of injury severity comprises from tearing a few muscle fibers from the connective tissue (aponeurosis and fascia) to a complete tear of the muscle from the tendon [2, 3]. The injury occurs in the muscle tissue, most commonly nearby or at the myotendinous junction (MTJ), the connection between muscle and tendon (aponeurosis). Therefore, the injury affects both the muscle and connective tissues [5, 6]. The injuries can lead to prolonged absence from sports and often result in recurrent injuries when training is resumed [7]. The rehabilitation of the affected muscle group following injury aims to expedite the recovery process and lower the likelihood of subsequent injuries. It remains, however, unknown whether the regenerating damaged tissue will fully resolve after rehabilitation.

This review aims to examine the effect of resistance training on recovery from acute muscle strains in the lower extremity and focuses on the outcomes: (1) time from injury to return to sport (RTS), (2) frequency of recurrent injuries/reruptures, and (3) structural alterations after acute muscle strains. A complete overview over all studies included in this review is found in the Supplementary Material Table S1.

### 1.1. Effect of Intervention on Return to Sport

Two studies by Askling et al. [8, 9] have demonstrated a superior effect of eccentric resistance training (L-protocol, relatively longer muscle length) compared to conventional concentric resistance training (C-protocol, relatively shorter muscle length) when it comes to the time to RTS. In the Askling studies, the L-protocol reduced the time to RTS by 45% and 38%, respectively. The incidence of reinjuries was low across all intervention groups, ranging from 0% to 7.14% after six months, and no significant differences between groups were observed. Other RCTs have, however, not found any significant differences between different rehabilitation interventions with a resistance training component on RTS sherry [10–14].

Bayer et al. [15, 16] compared the effects of early and delayed initiation of rehabilitation on RTS and reinjury rate. Amateur athletes with acute muscle strains in the thigh or calf verified by both ultrasound and MRI were included. The group that began early rehabilitation two days after the injury had a shorter time to return to sport (63 days) compared to the group that started rehabilitation nine days after the injury (83 days). Both groups followed a rehabilitation program consisting of four steps, including static stretching, isometric strength training, dynamic resistance training, and functional exercises including exercises with repeated sprints, cutting movements, and jumping combined with heavy resistance training. Return to sport was defined as the day when the athlete could fully participate in their sport without any symptoms and complete a functional test (repeated maximal sprints and single-leg jumps) with a maximum score of 1 on the pain numeric rating scale (NRS, from 0 = zero pain to 10 = worst imaginable pain). After 12 months, the rate of reinjury was similar in the two groups (0% for the delayed rehabilitation group and 5% for the early rehabilitation group).

### 1.2. Effect of Intervention on Rerupture Rate

In general, the risk of reruptures varied from 0% in some intervention groups—specifically in the L-protocol and late rehabilitation groups in the before mentioned Askling and Bayer studies—to 70% in the group applying static stretching, isolated progressive hamstring resistance exercise, and icing (“STST”) group in Sherry 2004. Sherry 2004 [11] and Mendiguchia 2017 [10]found a significant difference in the rate of reruptures between their two intervention groups as outlined below. Note that the reinjury rate was assessed after 12 months in five studies (including Sherry 2004), while it was assessed after only six months in the remaining three studies (including Mendiguchia 2017) [10, 11].

Sherry 2004 [11] included 24 participants with acute hamstring ruptures, Craig's grade 1-2 (partially muscle rupture, see supplementary Table S2). All participants engaged in sports that involved running and sprinting. The participants were stratified according to age and sex and were assigned to either the “STST” rehabilitation program (static stretching, isolated progressive hamstring resistance exercise, and icing) or the “PATS” (progressive agility training and trunk stabilizing) program. No significant differences in RTS were found between the two rehabilitation groups (37 days in the STST group and 22 days in the PATS group), and RTS defined as regained full strength and function while being pain-free [11]. The reinjuries rate was, however, significantly lower in the PATS group with 0 recurrences after 14 days and 1 recurrence after one year, compared to 6 in the STST group after 14 days and a total of 7 after one year.

Mendiguchia 2017 [10] randomized participants to either the general rehabilitation protocol (“RP program,” resembling the Askling L-protocol) or the rehabilitation algorithm (“RA program”). The algorithm customized rehabilitation for each participant, allowing the individuals to progress to the next step based on their individual status. The program included manual therapy, flexibility training, strength training, and agility exercises. In the general rehabilitation protocol, RTS was defined as the absence of all clinical signs of injury and successful completion of the Askling H-test. In the rehabilitation algorithm, RTS was defined when the athlete was able to fully participate in his/her sport. There was no significant difference in RTS between the groups; however, the rate of reinjury after six months was significantly lower in the rehabilitation algorithm group with 4%, compared to 25% in the general rehabilitation protocol group. It is worth noting that Mendiguchia 2017 applied different criteria for RTS between the two rehabilitation programs [10].

**Figure 1:**
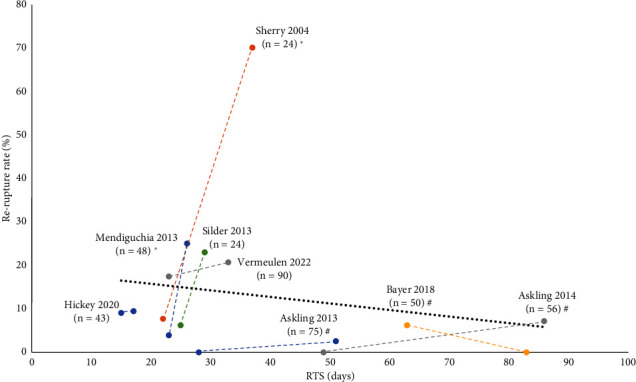
The relationship between days to return to sport (RTS) and the risk of subsequent reruptures. Each study is marked with two data points representing each rehabilitation group with the time until RTS on the *x*-axis and the rerupture rate (in %) in the *y*-axis. The two points representing two intervention groups are connected by a dotted line for clarity. *n* indicates the total number of participants in each study, and the trend line (mean of the two rehabilitation groups for each study) is indicated by a black dotted line. ^∗^Significant difference between intervention groups regarding RTS. #Significant difference between intervention groups regarding rerupture rate.

Randomized controlled trials by Silder 2013 [12], Hickey 2020 [13], and Vermeulen 2022 [14] did not show any differences in the time to RTS nor the number of reinjuries between intervention groups. Overall, a relatively short time to RTS (15–33 days) and a moderate proportion of reinjuries (6–21%) were observed compared to the other studies (Figure 1).

Silder 2013 [12] compared the effect of progressive running training and eccentric strength training (“PRES”) with the “PATS” program, which was identical to that used in Sherry 2004 [11]. Hickey 2020 [13] investigated the effect of rehabilitation within pain threshold limits NRS 4 versus pain-free rehabilitation. Vermeulen 2022 [14] compared two rehabilitation programs, where Askling L-protocol exercises were introduced on either day one of rehabilitation program (day five after injury) or later, when the participant could run without pain at 70% of their maximum running speed (on average day 16 after injury).

### 1.3. Structural Tissue Changes

While Sherry 2004 [11] relied solely on the clinical presentation for inclusion and follow-up of participants, the other RCTs included in this review additionally utilized diagnostic imaging. Askling concluded that there was a significantly shorter time to RTS for MRI-negative (i.e., no visible signs of damage on an MRI scan) participants compared to the MRI-positive group. In addition, a significant correlation was found between greater craniocaudal length of the injury, greater distance to the ischial tuberosity, involvement of the tendon, and longer time to RTS [8, 9].

Like Askling, Silder found a correlation between craniocaudal length, distance to the ischial tuberosity, and RTS [12]. Bayer on the contrary did not find any association between injury severity on MRI and the time to RTS but significant muscle atrophy six months postinjury, also observed by Silder 2013 [12, 16].

With respect to the fascicle length, Hickey 2020 saw an improvement from their initial clinical assessment at the time of a hamstring strain injury to RTS. In this study, fascicle length in the biceps femoris long head became significantly longer with rehabilitation with no difference between the groups at RTS. Fascicles were longer in the group training with pain up to 4 on the NRS at 2 months follow-up measurement [13].

In a study on individuals with a medial gastrocnemius strain injury, Busk-Nielsen et al. [17] reported significantly shorter fascicle length at the site of the injury compared to the uninjured contralateral side. Note that the data collection was performed several months to years after the injury. Furthermore, the authors reported a curvilinear fascicle shape during a unilateral dynamic movement, which indicate a lack of muscle fiber contractility in the previously affected muscle. The same study described additionally persisting structural changes to the deep aponeurosis (significantly enlarged) at the site of the injury (distal medial gastrocnemius) expanding long into the mid-belly portion of the gastrocnemius. Also function of the aponeurosis appears to be disturbed long time poststrain injury. Similar observations were recently made by Lazarczuk [18] in the biceps femoris long head. The authors report significantly smaller muscle-to-aponeurosis volume ratios and larger proximal aponeurosis volumes compared to noninjured hamstrings of a control group. These findings strongly support the note that there is a significant component of the connective tissue (i.e., the aponeurosis and thereby also the myotendinous junction), which is affected by the strain injury and seemingly not recovering completely over time [17].

The myotendinous junction (MTJ) is a highly specialized region where the muscle fibers integrate with the dense collagen fibrils at the tendinous side. There is extensive folding to increase the surface area [5] and a specific set of proteins present at the MTJ [19], most likely conferring stability to the junction subjected to high stress. It can be assumed that activity on both sides of the junction is required to restore the muscle-tendon link after injury disruption but very little is known about the capacity of each side to adapt. Interestingly, the tendinous side of the MTJ does not appear to be as collagen dense and well-ordered poststrain injury compared to a healthy MTJ, again underlying the connective tissue component in the incomplete repair of tissue structures after a strain injury [20]. The observations on the lack of reformation of a dense collagen matrix at the MTJ postinjury have been made on ultrasound-guided biopsies taken from the site of a previous strain injury with clear hypo-/hyperechoic areas on the US images. These biopsies further revealed marked fatty infiltration intra- and intermuscular, pathological signs in the muscle fibers such as mitochondrial accumulation subsarcolemma, sarcomere disruption, and a lack of sarcomeres in intact muscle fibers months to years after the injury [20]. Together, it seems thus that both the tendon and muscle side of the MTJ have difficulty in restoring the interface region to its preinjury state.

## 2. Discussion

### 2.1. Return to Sport

When examining RCT studies available, two factors have been identified, which significantly reduce the time to RTS. Firstly, eccentric exercises performed at long muscle lengths', as demonstrated in Askling 2013 [8] and Askling 2014 [9], have been found to accelerate RTS. Secondly, early initiation of rehabilitation, as illustrated in the Bayer study [15, 16], significantly shortens the time to RTS. There is a considerable variation when comparing RTS across studies from RTS 51–86 days in the C-protocol groups in Askling 2013 and 2014 [8, 9], respectively, while RTS was 28 and 49 days in the L-protocol groups in Askling 2013 and 2014, respectively. This significant variance in RTS highlights the importance of considering baseline characteristics of participants, which appears to be the most significant difference between those two studies. The basis for training at long muscle fiber lengths in Askling 2013 and 2014, i.e., the lengthening protocol [8, 9], stems from two studies trying to imitate the movement that led to the injury [21, 22]. Eccentric training is at the same time associated with the possibility to increase resting fascicle length [23, 24], and increased resting fascicle length is associated with a reduced hamstring injury risk [25]. However, longer fascicle length does not generally reduce the risk of strain injury for all types of athletes [26].

Interestingly, despite employing similar designs and interventions, the football players in Askling 2013 [8] exhibited markedly faster RTS compared to the sprinters and jumpers in Askling 2014 [9], which may indicate that football players may have returned to sport without fully restoring their function. This hypothesis is unproven. Bayer [15, 16] investigated the effect of early onset of rehabilitation (day two postinjury) compared to delayed onset of rehabilitation (day nine postinjury). A delay in rehabilitation means a longer period of reduced mobilization and thus an increased risk of structural and functional deficits of both muscle and tendon/aponeurosis. A study has shown that just 14 days of immobilization resulted in a significant loss of both muscle strength and reduced cross-sectional area [27]. By delaying the start of rehabilitation, a similar loss could occur in the injured athletes. However, Bayer found no difference in muscle mass or strength between the two intervention groups at RTS. In this context, it is important to point out that none of the studies have measured the strength and mechanical properties directly of the involved tendon/aponeurosis following a strain injury.

In general, there is a large variation in RTS between studies, and several factors can explain the variation such as the participants' baseline characteristics, injury severity, and criteria for returning to their sport. Regarding the participants' baseline characteristics, it is suggested that age may influence the injury prognosis as age *per se* has been identified as a risk factor of sustaining strain injuries [28], and aging is associated with a decrease in regenerative capacity [29]. In the studies included in this review, the participants' average age is 25 years, ranging from participants being on average five years younger [9] and on average eight years older [15, 16]. However, while participants in the Bayer study on average have the longest time to RTS, participants in the Askling 2014 study have the second longest time to RTS on average despite their younger age.

The level at which participants engage in their sport should also be considered, as it can be assumed that semiprofessional athletes have a better physical basis compared to recreational athletes. However, this hypothesis can be questioned, as the studies that included semiprofessional athletes found either some of the longest (Askling et al., 2013 and 2014) or some of the shortest time to RTS [10]. It is possible that the participants' baseline characteristics in combination with other factors not discussed so far play an important role in the prognosis for returning to sport. All RCT studies had to meet the requirement of having some degrees of structural damage or equivalent in relation to the severity of the injuries. However, in the Hickey 2020 study [13], no specific requirements for the severity of the injury were described. The Hickey study stands out by a short time to RTS and a relatively low risk of rerupture, which might be a direct result to less severe injuries compared to the other studies. Both Mendiguchia 2017 [10] and Vermeulen 2022 [14] used the same classification system (Peetron's) [30]. Mendiguchia included grade 1 injuries, while Vermeulen included grade 1-2 injuries. When comparing the two studies, the Vermeulen study had longer time to RTS, and higher risk of rerupture compared to Mendiguchia 2017. In the Askling studies [8, 9], a small subgroup of participants with MRI-negative injuries were included, and their RTS was found to be six and 15 days, respectively. In comparison, the MRI-positive group had an RTS of 23 and 45 days, respectively. These findings strongly support the hypothesis that more severe injuries require a longer time to RTS and that MRI-positive injuries are more severe than MRI-negative injuries.

### 2.2. Rerupture Rate

The time span during which reinjuries were recorded varied between six and 12 months across studies. The average risk of reinjuries in the six months follow-up group is 9% and the average risk in the 12 months follow-up group is 15%, indicating a temporal aspect. Generally, reinjuries primarily occur in the early stages after RTS: Sherry 2004 [11], Silder 2013 [12], and Vermuelen 2022 [14], which is supported by previous studies showing the same tendency for early reinjuries, including Wangensteen 2016 [31], with a median time from RTS to reinjury of 24 days among 19 athletes with reinjuries after acute hamstring muscle ruptures. Sherry 2004 [11] and Mendiguchia 2017 [10] demonstrated statistically significant differences in the rate of reruptures between their two intervention groups, and it is notable that these two studies also show the highest proportion of reinjuries in the intervention groups with the longer time to RTS. These findings indicate that the risk of reruptures is not exclusively reduced by delaying the time to RTS but suggests that strain injuries require a rigorous and specific rehabilitation program. At the same time, it should be noted that the rehabilitation group with the highest rerupture rate in each study (70% in Sherry and 25% in Mendiguchia) are the two groups with the highest rerupture rate overall in this review.

The studies without a statistical difference between intervention groups in reinjury might reflect a generally lower risk of reinjury in scientific projects as a result to well-designed and tightly supervised rehabilitation programs and appropriate criteria for RTS. This statement is supported also by observations by Tyler et al. [32] that demonstrated that reinjuries only occurred in the group on noncompliant athletes to a rehabilitation regimen. In addition, some studies include a low number of participants and might therefore be underpowered to detect significant differences unless there is a large intervention effect. The high rate of reinjury observed in one of the intervention groups in the Sherry and Mendiguchia studies may be attributed to premature RTS, potentially before adequate healing of the involved tissues. However, when comparing the studies, the criteria do not seem to differ significantly from the common criteria. The Mendiguchia rehabilitation algorithm group [10] used Askling's [8, 9] criteria for RTS. Nevertheless, Mendiguchia's study observed a significant proportion of reruptures (25%) compared to Askling's studies (0% in both L-protocol groups). Mendiguchia points out that grade 1 muscle ruptures (based on Peetron's classification system [30]) pose the greatest risk of rerupture, and that only type 1 injuries were included in the Mendiguchia study. However, there is limited evidence to support that grade 1 injuries have a higher rate of rerupture than grade 0 or grade 2 injuries [7]. Overall, these findings emphasize a relationship between the severity of the injury and the risk of resrupture. At the same time, the cohort study shows a relationship between the severity of the injury and the time to RTS. For injuries of grades 1–4, RTS occurred at 7, 12, 25, and 55 days [33]. When comparing injury severity between studies, it is important to note that different frameworks and facilitators of studies affect outcomes and contribute to varying results in this example and in general.

### 2.3. Relation between Time to RTS and Reinjury Rate Postinjury

**Figure 2:**
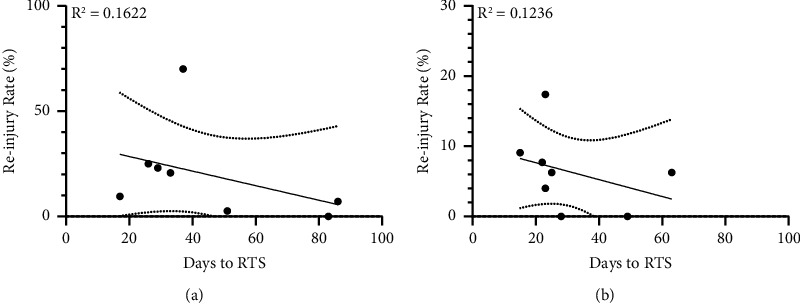
The relationship between days to return to sport (RTS) and the risk of subsequent reruptures in the rehabilitation groups with the respective highest RTS within each study (a) and the relationship between days to return to sport (RTS) and the risk of subsequent reruptures in the rehabilitation groups with the respective lowest RTS within each study (b).

The relationship between time to RTS and reinjury rate appears to be inversely proportional, as indicated in Figures 1, 2(a), and 2(b) by declining linear trend lines (*a* < 0). Studies with a longer time to RTS show a tendency towards a lower rerupture rate. This tendency is most obvious in Figure 2(a) which displays the relationship between RTS and reruptures in the rehabilitation groups with the higher RTS for each study. In Figure 2(b), this tendency seems to be faded by a flattening trendline, for the rehabilitation groups with the lower RTS. This pattern indicates that these rehabilitation programs, which were successful in shortening the time to RTS, were also favorable in reducing the risk of reruptures. This relation differs from study to study but can overall be visualized in Figure 1 by *a* being >0 in the linear functions connecting corresponding data points. Furthermore, the pattern challenges the prevailing assumption that a longer time to RTS lowers the risk of reinjury *per se*. In contrast, it is noteworthy to observe that the most effective rehabilitation programs successfully enhance both outcomes.

### 2.4. Structural Changes Postinjury

Structural tissue changes, such as a defect in muscle fiber continuity and insertion in the tendon/aponeurosis, occur not only immediately after an injury but also persist for a long time thereafter. Accumulating data suggests that tissue changes as muscle atrophy and fatty infiltration probably develop over time postinjury and are irreversible [20]. As the loss of muscle mass did not improve from three to six months poststrain injury despite full activity following RTS there likely is a permanent loss of muscle mass following strain injuries. Muscle atrophy following strain injuries is proposed to initially be caused by a rupture between the contractile elements and the connected tendon/aponeurosis with focal immobilization of the affected muscle fibers. Recent data do not suggest that the contractility of the affected muscle fibers is restored as the pattern of fascicle behavior during dynamic movement suggests that the fibers are pulled along by the surrounding tissue instead of contracting and thereby shortening as observed in unaffected muscle tissue. Furthermore, there is accumulating evidence that the aponeurosis following a strain injury is altered in structure and most likely also function, which also is not restored over time.

While some studies found an association of structural abnormalities on MRI scans with time to RTS [8, 9, 12], other studies did not find a link between imaging and RTS. A thorough study by Wangensteen et al. [34] investigated whether MRI-based injury severity grading would be able to predict time to RTS and found no predictability by using MRI grading. Interestingly, long-term structural abnormalities suggest a discrepancy between the subjective sensation of being injury-free, muscle function measured by physical tests, and the state of the damaged tissue at RTS and thereafter. However, one study on chronic strain injuries examined amateur athletes subjectively judging their previously injured limb compared to the contralateral healthy side. There were drastically lower scores translating into less function, more pain, and less confidence in their previously injured muscles. Although scores improved slightly with rehabilitative measures, they remained significantly lower in the injured limbs compared to the uninjured limbs [20].

## 3. Conclusion

The studies reviewed show varying time to return to sport (15 to 86 days) and rerupture rates (0% to 70%). Injury severity may correlate with longer return times and lower rerupture risks. Although there are limited studies on the subject, there is evidence to suggest that earlier rehabilitation and training at long muscle lengths may lead to a shorter time to return to sport. Delaying the time to RTS may potentially reduce the risk of reinjury for both amateur and professional athletes, but only if the athletes comply with an appropriate rehabilitation program. However, an unnecessary extension of the rehabilitation period could lead to significant career and financial losses for the professional athlete, as would a rerupture. For recreational athletes, a long RTS or a high rerupture rate might mean discontinuation with physical activity which, in general, is not considered a health-promoting concept. Gradually transitioning from rehabilitation to full participation in sports most likely prevents an unnecessarily long rehabilitation period and reduces the risk of reinjuries. A continued tertiary injury prevention effort is also likely to reduce the risk of reinjury after return to sport. There are significant structural tissue changes after a muscle strain injury, which affects both muscle tissue with fatty infiltration, disorganized sarcomeres, and noncontracting fascicles and the connective tissue. Data presented in recent years strongly suggest that these tissue changes are persisting and to some degree probably irreversible.

## Data Availability

The literature data supporting this narrative review are from previously reported studies and datasets, which have been cited. The processed data are available from the corresponding author upon request.

## Supplementary Materials

Supplementary Materials Supplementary Table S1: complete overview over studies included in this review with methods and number of participants, injury type and grading, interventions, and short summary of results. Supplementary Table S2: grading according to classification systems. Overview over studies included in this review and grading systems applied in these different studies.

## References

[1] Edouard P., Branco P., Alonso J. M. (2016). Muscle injury is the principal injury type and hamstring muscle injury is the first injury diagnosis during top-level international athletics championships between 2007 and 2015. *British Journal of Sports Medicine*.

[2] Patel A., Chakraverty J., Pollock N., Chakraverty R., Suokas A. K., James S. L. (2015). British athletics muscle injury classification: a reliability study for a new grading system. *Clinical Radiology*.

[3] Mueller-Wohlfahrt H. W., Haensel L., Mithoefer K. (2013). Terminology and classification of muscle injuries in sport: the Munich consensus statement. *British Journal of Sports Medicine*.

[4] Noonan T. J., Garrett W. E. (1999). Muscle strain injury: diagnosis and treatment. *Journal of the American Academy of Orthopaedic Surgeons*.

[5] Knudsen A. B., Larsen M., Mackey A. L. (2015). The human myotendinous junction: an ultrastructural and 3D analysis study. *Scandinavian Journal of Medicine & Science in Sports*.

[6] Tidball J. G., Salem G., Zernicke R. (1993). Site and mechanical conditions for failure of skeletal muscle in experimental strain injuries. *Journal of Applied Physiology*.

[7] de Visser H. M., Reijman M., Heijboer M. P., Bos P. K. (2012). Risk factors of recurrent hamstring injuries: a systematic review. *British Journal of Sports Medicine*.

[8] Askling C. M., Tengvar M., Thorstensson A. (2013). Acute hamstring injuries in Swedish elite football: a prospective randomised controlled clinical trial comparing two rehabilitation protocols. *British Journal of Sports Medicine*.

[9] Askling C. M., Tengvar M., Tarassova O., Thorstensson A. (2014). Acute hamstring injuries in Swedish elite sprinters and jumpers: a prospective randomised controlled clinical trial comparing two rehabilitation protocols. *British Journal of Sports Medicine*.

[10] Mendiguchia J., Martinez-Ruiz E., Edouard P. (2017). A multifactorial, criteria-based progressive algorithm for hamstring injury treatment. *Medicine & Science in Sports & Exercise*.

[11] Sherry M. A., Best T. M. (2004). A comparison of 2 rehabilitation programs in the treatment of acute hamstring strains. *Journal of Orthopaedic & Sports Physical Therapy*.

[12] Silder A., Sherry M. A., Sanfilippo J., Tuite M. J., Hetzel S. J., Heiderscheit B. C. (2013). Clinical and morphological changes following 2 rehabilitation programs for acute hamstring strain injuries: a randomized clinical trial. *Journal of Orthopaedic & Sports Physical Therapy*.

[13] Hickey J. T., Timmins R. G., Maniar N. (2020). Pain-free versus pain-threshold rehabilitation following acute hamstring strain injury: a randomized controlled trial. *Journal of Orthopaedic & Sports Physical Therapy*.

[14] Vermeulen R., Whiteley R., van der Made A. D. (2022). Early versus delayed lengthening exercises for acute hamstring injury in male athletes: a randomised controlled clinical trial. *British Journal of Sports Medicine*.

[15] Bayer M. L., Magnusson S. P., Kjaer M. (2017). Early versus delayed rehabilitation after acute muscle injury. *New England Journal of Medicine*.

[16] Bayer M. L., Hoegberget-Kalisz M., Jensen M. H. (2018). Role of tissue perfusion, muscle strength recovery, and pain in rehabilitation after acute muscle strain injury: a randomized controlled trial comparing early and delayed rehabilitation. *Scandinavian Journal of Medicine & Science in Sports*.

[17] Nielsen L., Svensson R., Fredskild N. (2023). Chronic changes in muscle architecture and aponeurosis structure following calf muscle strain injuries. *Scandinavian Journal of Medicine & Science in Sports*.

[18] Lazarczuk S. L., Collings T. J., Hams A. H. (2024). Biceps femoris long head muscle and aponeurosis geometry in males with and without a history of hamstring strain injury. *Scandinavian Journal of Medicine & Science in Sports*.

[19] Karlsen A., Gonzalez-Franquesa A., Jakobsen J. R. (2022). The proteomic profile of the human myotendinous junction. *iScience*.

[20] Bayer M. L., Hoegberget-Kalisz M., Svensson R. B. (2021). Chronic sequelae after muscle strain injuries: influence of heavy resistance training on functional and structural characteristics in a randomized controlled trial. *The American Journal of Sports Medicine*.

[21] Chumanov E. S., Heiderscheit B. C., Thelen D. G. (2007). The effect of speed and influence of individual muscles on hamstring mechanics during the swing phase of sprinting. *Journal of Biomechanics*.

[22] Schache A. G., Dorn T. W., Blanch P. D., Brown N. A. T., Pandy M. G. (2012). Mechanics of the human hamstring muscles during sprinting. *Medicine & Science in Sports & Exercise*.

[23] Presland J. D., Timmins R. G., Bourne M. N., Williams M. D., Opar D. A. (2018). The effect of Nordic hamstring exercise training volume on biceps femoris long head architectural adaptation. *Scandinavian Journal of Medicine & Science in Sports*.

[24] Suskens J. J. M., Secondulfo L., Kiliç Ö (2023). Effect of two eccentric hamstring exercises on muscle architectural characteristics assessed with diffusion tensor MRI. *Scandinavian Journal of Medicine & Science in Sports*.

[25] Timmins R. G., Bourne M. N., Shield A. J., Williams M. D., Lorenzen C., Opar D. A. (2016). Short biceps femoris fascicles and eccentric knee flexor weakness increase the risk of hamstring injury in elite football (soccer): a prospective cohort study. *British Journal of Sports Medicine*.

[26] Lee Dow C., Timmins R. G., Ruddy J. D. (2021). Prediction of hamstring injuries in Australian football using biceps femoris architectural risk factors derived from soccer. *The American Journal of Sports Medicine*.

[27] de Boer M. D., Maganaris C. N., Seynnes O. R., Rennie M. J., Narici M. V. (2007). Time course of muscular, neural and tendinous adaptations to 23 day unilateral lower-limb suspension in young men. *The Journal of Physiology*.

[28] Gabbe B. J., Bennell K. L., Finch C. F., Wajswelner H., Orchard J. W. (2006). Predictors of hamstring injury at the elite level of Australian football. *Scandinavian Journal of Medicine & Science in Sports*.

[29] Muñoz-Cánoves P., Neves J., Sousa-Victor P. (2020). Understanding muscle regenerative decline with aging: new approaches to bring back youthfulness to aged stem cells. *FEBS Journal*.

[30] Peetrons P. (2002). Ultrasound of muscles. *European Radiology*.

[31] Wangensteen A., Tol J. L., Witvrouw E. (2016). Hamstring reinjuries occur at the same location and early after return to sport: a descriptive study of MRI-confirmed reinjuries. *The American Journal of Sports Medicine*.

[32] Tyler T. F., Schmitt B. M., Nicholas S. J., McHugh M. P. (2017). Rehabilitation after hamstring-strain injury emphasizing eccentric strengthening at long muscle lengths: results of long-term follow-up. *Journal of Sport Rehabilitation*.

[33] Malliaropoulos N., Isinkaye T., Tsitas K., Maffulli N. (2011). Reinjury after acute posterior thigh muscle injuries in elite track and field athletes. *The American Journal of Sports Medicine*.

[34] Wangensteen A., Guermazi A., Tol J. L. (2018). New MRI muscle classification systems and associations with return to sport after acute hamstring injuries: a prospective study. *European Radiology*.

