# Supplementary material for: Return to Sport, Reinjury Rate, and Tissue Changes after Muscle Strain Injury: A Narrative Review
Source: Transl Sports Med. 2024 Sep 4;2024:2336376. doi: 10.1155/2024/2336376 (PMC11390226; doi:10.1155/2024/2336376)
Supplement: Supplementary Materials — Supplementary Table S1: complete overview over studies included in this review with methods and number of participants, injury type and grading, interventions, and short summary of results. Supplementary Table S2: grading according to classification systems. Overview over studies included in this review and grading systems applied in these different studies. [file 2336376.f1.docx]

**Supplementary data**

**Return to sport, re-injury rate and tissue changes after muscle strain injury: A narrative Review**

Mette W Wulff^1,2^, Abigail L Mackey^1,2^, Michael Kjær, ^1,2^, Monika L Bayer^1,2^

Author affiliations

^1^Institute of Sports Medicine Copenhagen, Department of Orthopedic Surgery M, Copenhagen University Hospital – Bispebjerg Frederiksberg, Copenhagen, DK

^2^Center for Healthy Aging, Faculty of Health and Medical Sciences, University of Copenhagen, Copenhagen, DK

**Supplementary table S1.**

Overview over studies included in this review

| Article | Methods + participant count | Injury type + grading | Intervention | Results |
| --- | --- | --- | --- | --- |
| Sherry et al.  2004 | RCT*  (24) | Hamstring injuries.  Grade 1-2 (Craig’s classification); full ruptures not included. | Stretching and resistance training (STST) vs. progressive agility and trunk-stabilizing exercises (PATS). | The PATS group had fewer re-ruptures than the STST group. No significant difference observed in terms of return to sport (RTS) between the groups. |
| Silder et al.  2013 | RCT  (29) | Hamstring injuries.  Full ruptures not included. | Progressive running and eccentric strength training (PRES) vs. progressive agility and trunk-stabilizing exercises (PATS). | No significant difference observed in terms of RTS or re-ruptures between the two groups.  All participants had structural changes at RTS (on MRI). |
| Askling et al.  2013 | RCT  (75) ◊ | Hamstring injuries.  Grading is not further described. | Conventional exercises (C-protocol) vs. lengthening exercises (L-protocol). | L-protocol shortened the time to RTS. Re-injury rate was low in both groups with no significant difference between groups. |
| Askling et al.  2014 | RCT  (56) ◊ | As Askling 2013. | As Askling 2013. | As Askling 2013. |
| Mendiguchia et al.  2017 | RCT  (48) ◊ | Hamstring injuries.  Peetron’s grade 1.  A maximum of 5% involvement of the muscle. | General rehabilitation (RP) vs. rehabilitation algorithm (RA). | Re-injury rate was lower in the RA-group. No significant difference in RTS between groups. |

| Bayer et al.  2017 | RCT  (50) | Thigh- or calf muscle injuries.  Grade 3-4 (Munich consensus statement) | Early (2 days after injury) vs. delayed (9 days after injury) rehabilitation. | Early rehabilitation reduced RTS by 20 days.  No significant difference between groups in terms of re-rupture. |
| --- | --- | --- | --- | --- |
| Bayer et al.  2018 | As Bayer 2017 | As Bayer 2017 | As Bayer 2017 | DCE-MRI showed structural changes in both groups with muscle atrophy after 3 and 6 months. Muscle function was similar in the groups. |
| Hickey et al.  2020 | RCT  (43) | Hamstring injuries.  Not graded. | Pain-free vs. pain-threshold (NRS 4) rehabilitation. | No significant difference in terms of RTS or re-injuries.  Increased strength and biceps femoris fascicle length in the pain-threshold group. |
| Vermeulen et al.  2022 | RCT  (90) | Hamstring injuries.  Peetron’s grad 1-2. A maximum of 50% involvement of the muscle. | Early rehabilitation (day 1 after injury) vs. delayed rehabilitation (when being able to run at 70% of maximum running speed). | No significant difference observed in terms of RTS or re-ruptures between the two groups. |

*RCT: *Randomized controlled trial*

**RTS: *Return to sport*

◊: (semi)professional athletes

**Table S2. Grading according to classification systems**

| **Study** | **Classification system** | **Grading** | **Description** |
| --- | --- | --- | --- |
| **Sherry 2004** | Craig’s | Grade 1-2 | Partially muscle rupture (no full ruptures) (15). |
| **Silder 2013** | *Not specified* | *Not specified* | All grades apart from total ruptures. |
| **Askling 2013** | *Not specified* | *Not specified* | MRI verified strains; all grades included. |
| **Askling 2014** | *Not specified* | *Not specified* | MRI verified strains; all grades included. |
| **Mendiguchia 2017** | Peetron’s | Grade 1 | Lesion on US, up to 5% (or 1 cm) of the muscle involved (19). |
| **Bayer 2018** | Munich consensus statement | Grade 3-4 | US and MRI verified strains, partial to (sub)total ruptures (3). |
| **Hickey 2020** | *Not specified* | *Not specified* | All grades apart from total ruptures. |
| **Vermeulen 2022** | Peetron’s | Grade 1-2 | Lesion on US: up to 50% of the muscle involved (19) |
